# Training auscultatory skills: computer simulated heart sounds or additional bedside training? A randomized trial on third-year medical students

**DOI:** 10.1186/1472-6920-10-3

**Published:** 2010-01-18

**Authors:** Øystein Sverdrup, Torstein Jensen, Svein Solheim, Knut Gjesdal

**Affiliations:** 1 Faculty of Medicine, University of Oslo, Oslo, Norway; 2 Lovisenberg Deaconess Hospital, Oslo, Norway; 3 Department of Cardiology, Ullevål University Hospital, Oslo, Norway

## Abstract

**Background:**

The present study compares the value of additional use of computer simulated heart sounds, to conventional bedside auscultation training, on the cardiac auscultation skills of 3^rd ^year medical students at Oslo University Medical School.

**Methods:**

In addition to their usual curriculum courses, groups of seven students each were randomized to receive four hours of additional auscultation training either employing a computer simulator system or adding on more conventional bedside training. Cardiac auscultation skills were afterwards tested using live patients. Each student gave a written description of the auscultation findings in four selected patients, and was rewarded from 0-10 points for each patient. Differences between the two study groups were evaluated using student's t-test.

**Results:**

At the auscultation test no significant difference in mean score was found between the students who had used additional computer based sound simulation compared to additional bedside training.

**Conclusions:**

Students at an early stage of their cardiology training demonstrated equal performance of cardiac auscultation whether they had received an additional short auscultation course based on computer simulated training, or had had additional bedside training.

## Background

Proficiency in cardiac auscultation has been and still is an important skill in clinical medicine. Students often find it difficult to determine the timing and characterization of the heart sounds. Several surveys attest to the lack of cardiac auscultation skills among medical students [1] as well as among clinicians [1-3].

The development of devices to facilitate learning of auscultation started already in the late 1960's. Mannequins, electronic stethoscopes, computer programs and simulators have since then been developed and marketed. The first mannequin made for teaching students heart auscultation was introduced in 1968 and named "Harvey" [4]. In 1987 a study on the use of Harvey on fourth-year medical students showed improved skills compared to a control group not using the device [5]. In another trial that tested modern equipment like electrophonogram and infrared stethoscope [6], the authors concluded that such equipment is a valuable supplement to conventional bedside training. Although many reports attest to the value of using teaching devices for auscultation training, studies usually compare such training with no additional training [6,7]. It is still unclear whether teaching programs with technical devices facilitate learning skills because they are more effective, or if the benefits merely are due to more time spent on the subject.

CardioSim is a simulator of heart sounds designed and manufactured by Cardionics Inc., (Webster, Texas, USA). The equipment comprises a simulator, loudspeakers, a pulse generator, and as an additional option, a "Simulscope unit" that permits simultaneous listening through an infrared sound system for stethoscopes. The phonograms and ECGs are displayed on a computer screen. A manual/short text can be provided. Some of the sounds are also presented with animations of the heart and the blood flow. Possibly, the program of computer-generated sounds and murmurs might facilitate learning due to its didactic approach. Alternatively, the auscultation of patients might be better, mimicking clinical real life. We have previously shown that the use of electronic stethoscopes did not augment students' performance in auscultation skills [8]. In the present study we have used a similar study design and compared two brief courses of additional auscultation training: one with additional conventional bedside training and another using the CardioSim Auscultation System (CAS). The outcome was the students' ability to describe and interpret cardiac sounds and murmurs in patients.

## Methods

### The ordinary teaching program for cardiac auscultation in Oslo

Auscultation training of medical students at the University of Oslo (Oslo, Norway) starts with a brief introduction by a course in clinical examination in the second of six years of study. A trainee doctor in internal medicine or surgery supervises groups of six to eight students. Over two to four hours the basics are taught by auscultation of peers and selected patients. In the third year students are given a two hours lecture on the use of the stethoscope in cardiology, followed by bedside two-hours training sessions, and at least one session focuses on cardiac auscultation. During practice in the emergency department and in the wards, students examine patients and make reports, and they are responsible for obtaining feed-back on their findings from the physicians on duty. At the end of the 3^rd ^year there is a clinical exam, and the students must demonstrate their skills in taking history and performing clinical examination. Later on, no curriculum course is dedicated solely to auscultation, but the subject will be in focus of the clinical teaching whenever relevant.

### Study subjects

The trial was conducted at Ullevål University Hospital in 2007. Forty-nine 3^rd ^year medical students were divided into seven teaching groups. The groups were randomized to receive additional auscultation training with the CardioSim Auscultation System (CAS) (4 groups, 28 students), or to conventional bedside training (3 groups, 21 students). All students also received regular training as defined in the curriculum. The present intervention study started when they had finished the main cardiology part of the term.

### Design of the study

For the purpose of the present study, each student group participated in two teaching sessions, each of two hours, in addition to the regular teaching program. First, all students had a two hour training session with one of two instructors (TJ, SS), both trainee doctors in cardiology and experienced teachers. The instructional objectives were that students should be able to describe the heart sounds, the timing, location, quality and grading of murmurs, and provide a reasonable clinical diagnosis based upon their findings. One group had bedside training and the other used the CardioSim Auscultation System, with a training program selected from the menu of CAS. Approximately one week later both groups had another two hours session, now without the teacher. The CAS groups were instructed to follow a pathway through the computer menu, exposing them to a number of common and less common heart sounds and murmurs, with descriptions, phonograms and occasionally, drawings or short videos that demonstrated the sounds and the mechanisms involved. Discussion was recommended, and they should not move on to the next case until all agreed. The groups allocated to bedside training received a list of suitable patients from the wards, and the students had access to the physicians' descriptions of the heart sounds and murmurs. Training was finished within 12 weeks. The intervention courses started after the main cardiology teaching of the term had taken place, and their main curriculum topics during this period were pulmonary and renal pathophysiology and clinics.

#### Test of auscultation skills

The outcome was students' performance in a clinical exam, during which each student auscultated four different patients with heart murmurs or other pathological findings related to the heart. The students were not allowed to request clinical information, and discussion was not permitted. After 10-15 minutes with each patient, they described the heart sounds and murmurs on a questionnaire, and eventually suggested a diagnosis for the patient. They also reported how much auscultation training they actually had experienced during this term, beyond the core curriculum training. Students were free to attend on any one of three exam days. Suitable patients were selected at the echocardiography laboratory. Seven patients participated in the examinations; two were available all three days, one participated for two days, and four participated for one day. Table 1 shows the principal diagnoses of the study patients.

**Table 1:** Cardiac diagnoses of seven patients who each participated for 1-3 days in the auscultation tests.

DAY	Test 1	Test 2	Test 3	Test 4
**1**	aortic stenosis	aortic regurgitation	pulmonary hypertension	mitral insufficiency

**2**	aortic stenosis	aortic regurgitation	mitral insufficiency plus aortic regurgitation	mitral insufficiency

**3**	aortic stenosis	aortic regurgitation	aortic stenosis	mitral insufficiency

Patients 1 and 2 are identical all three days. Patient 4 is the same on days 1 and 2. Each student was exposed to four patients.

#### Scoring

Two experienced cardiologists examined the patients and reached consensus on the correct description of the heart sounds, murmurs and the clinical diagnoses of the patients. The questionnaire was structured to test the students' proficiency in writing an auscultation report. Hence, most points were earned by writing a good report, rather than pinpointing the correct diagnosis. For each patient the student could get a maximum of ten points, giving a possible maximum score of 40 points. They were expected to report

1. a description of the heart sounds if abnormal.

2. if a murmur was detected, description of its timing (systolic/diastolic), maximal point, direction of the sound radiation, and grade and quality of the sound.

3. the assumed diagnosis.

#### Statistics

The null hypothesis was that the mean score of the auscultation test would not differ between the two intervention groups. The Student's t-test (two-sided) was used to compare the two groups, and a p < 0.05 was considered statistically significant. The Pearson test was used to examine if the time between teaching and testing were related to the outcome. We could not estimate the statistical power before the study, but performed a *post hoc-*analysis.

#### Ethics

According to the regulations for medical research in Norway this study protocol could be performed without any consideration or approval by the Regional Committee for Medical Research Ethics (ref 0910-01a Gjesdal).

## Results

All students but one from the CAS group attended the scheduled teaching programs. Five students from each group did not show up for the test. Thus, 37 of the students (75%) completed the trial, 21 students from the CAS groups and 16 from the bedside teaching groups. Data were found to be normally distributed. Mean score sum for four patients were 19.1 ± SD 5.3 (range 11 to 28) in the bedside group and 18.2 ± 7.0 (range 2 to 28) in the CAS group (p = 0.698). *Post hoc *analysis of the data showed that the difference in mean score would have had to be at least 6.1 to reach statistical significance between the groups.

Since only two test patients participated all three days, we analyzed the Students' test score on these separately: The mean scores ± SD then were 10.3 ± 3.6 (range 6-17) in the bedside group and 11.4 ± 5.1 (range 1-19) in the CAS group (p = 0.494).

### Protocol deviations and problems encountered during the study

Technical problems with the CAS equipment during the study led to a delay during the self study training for one group, another group had a less efficient session. Since teaching took place throughout the term, the time span between teaching and testing varied between the teaching groups. The mean difference from training to the exam was 6.0 weeks in the CAS group, and 5.3 weeks in the bedside group. The results were slightly better in students who had a brief time interval between the teaching and the test, but this was not a significant confounder (The Pearson correlation coefficient r = -0.24 between individual score sum and time interval (p = 0.16)).

## Discussion

In the present study, the students' performance in auscultation of cardiac patients was similar whether they had received additional teaching with the computer-based teaching program, or had additional traditional bedside training. The strengths of our trial include the randomized design, the similar amount of additional training in both groups, and the blinded questionnaire evaluation.

In a previous study by Horiszny [7], the ability to correctly interpret simulated heart sounds was better among those who had participated in the teaching session compared to those who did not. However, no other teaching form was offered to those who did not use the simulator, so the better results might be due to more time spent on learning auscultation or to higher motivation among those who attended the teaching session. Another randomized trial revealed advantages using classroom teaching compared to CD-ROM teaching among medical students [9]. In that trial the results were satisfying, but the students had difficulties in classifying murmurs and the second heart sound.

### Poor results in both groups

Generally the auscultation skills in the present study were poor. The best score achieved was 28 of 40 points - 70%. Possibly, the time allocated to auscultation training was too brief for obtaining the skills required to make a good auscultation report at this stage of their medical education. This observation is supported by other trials that have evaluated some of the same aspects, both in medical students and doctors [1].

We found a weak, non-significant negative correlation between the time interval from the last teaching session to the test. This is compatible with the common experience that following a short intensive course, knowledge and skills reach the short memory only. A controlled intervention study concluded that five hundred repetitions of four basic cardiac murmurs significantly improved medical students' proficiency in recognizing basic cardiac murmurs. The authors concluded that cardiac auscultation is, in part, a technical skill [10]. It is, however, hard to get acceptance for more time spent on auscultation training in the congested medical core curriculum.

### Limitations of the study

The technical problems with the CAS equipment limited the value of the computer-based training sessions. Our selection of program components from the CAS had not been tried previously, and might have been improved with more experience. Benefits from the CAS system might further have increased, had the equipment been more accessible for the students, or if we had used an auscultation mannequin in addition [11]. We had no pre-intervention test. Hence we cannot know for certain, the value of the additional training in any of the two arms. A more ideal design would have been to randomize the groups at the very first time the students were introduced to heart auscultation, and then keep the same groups throughout the medical education. However, such an approach would be complicated. It would demand at least 5 years follow-up of each student. Furthermore, the Faculty of Medicine in Oslo reorganizes the teaching groups from one term to another, in order to train the students to cope with new group settings. A final limitation to our approach is that we tested auscultation skills only; recently, benefit has been reported from additional teaching on complete cardiac examination with virtual patients [12].

We had expected to find advantages by using the simulator, since the teaching could introduce each concept step by step, and it allowed the students to carefully study the different cardiac murmurs. However, no demonstrable difference between use of the heart sound simulator and bedside training was found. This does not, however, exclude a value of the computer-assisted auscultation system. With shorter hospital stays and difficulty in faculty recruitment, bedside teaching is a challenge that simulation technology may help to overcome. Given that simulator practice is readily available to students, at variance from patients and instructors, simulator practice may be a reasonable alternative to bedside examination practice.

## Conclusions

In a randomised design students who had received an introductory course in cardiac auscultation, underwent an additional 4 hours course based on computer simulated training, or had additional bedside training. When tested on patients with heart murmurs, the two groups had equal performance with respect to sound and murmur description and diagnosis.

## Abbreviations

CAS: CardioSim Auscultation System;

## Competing interests

The authors declare that they have no competing interests.

## Authors' contributions

ØS organized the trial, participated in the study design, the acquisition of data, evaluated the results, performed the statistical analysis and drafted the manuscript. TJ led the teaching of the students, participated in the design of the study and organized the teaching program on the CAS. SS led the teaching of the students, participated in the design of the study and described the heart sounds for the test. KG conceived the study, organized the trial and the design, and supervised the project. All authors have contributed to manuscript writing, and all have read and approved the final manuscript

## Pre-publication history

The pre-publication history for this paper can be accessed here:

http://www.biomedcentral.com/1472-6920/10/3/prepub
